# The Use of Intravitreal Ranibizumab for Choroidal Neovascularization Associated with Vogt-Koyanagi-Harada Syndrome

**DOI:** 10.1155/2011/747648

**Published:** 2011-08-03

**Authors:** A. M. Kolomeyer, M. S. Roy, D. S. Chu

**Affiliations:** The Institute of Ophthalmology and Visual Science, University of Medicine and Dentistry of New Jersey, Newark, NJ 07103, USA

## Abstract

*Purpose*. To describe the use of intravitreal ranibizumab for choroidal neovascular membrane (CNVM) secondary to Vogt-Koyanagi-Harada (VKH) syndrome. *Methods*. Interventional case report. *Results*. A 50-year-old woman presented with conjunctival injection and bilateral eye pain. Vision was 20/400 and 20/80 in the right and left eyes, respectively. Bilateral iritis, vitritis, and choroidal thickening were evident. Exudative retinal detachment was present in the left eye. Corticosteroid treatment improved vision to 20/40 bilaterally. Methotrexate (MTX) was initiated and vision remained stable for 3 months. After a 5-month loss to follow-up, vision in the left eye decreased to finger counting (CF) and a parafoveal CNVM was identified. After 3 intravitreal ranibizumab injections, vision improved to 20/40. Twelve months later, despite inflammation control, vision decrease to CF due to recurrent CNVM. A fourth ranibizumab injection was given. Twenty months later, best-corrected vision was 20/400, and an inactive CNVM was present in the left eye. *Conclusion*. After initial CNVM regression and visual acuity improvement due to ranibizumab, the CNVM recurred and became refractory to treatment. Despite control of inflammation and neovascularization, VKH chronicity lead to permanent vision loss in our patient. A combinational treatment approach may be required in such patients.

## 1. Introduction

Vogt-Koyanagi-Harada syndrome (VKH), an autoimmune reaction against melanocytes [1], is characterized by granulomatous panuveitis, exudative retinal detachments (RD), subretinal fibrosis, choroidal neovascular membrane (CNVM) formation, and extraocular manifestations [2]. CNVM is found in 2–15% of VKH patients [3]. We report on VKH-associated subfoveal CNVM treated with intravitreal ranibizumab.

## 2. Case Report

**Figure 1:**
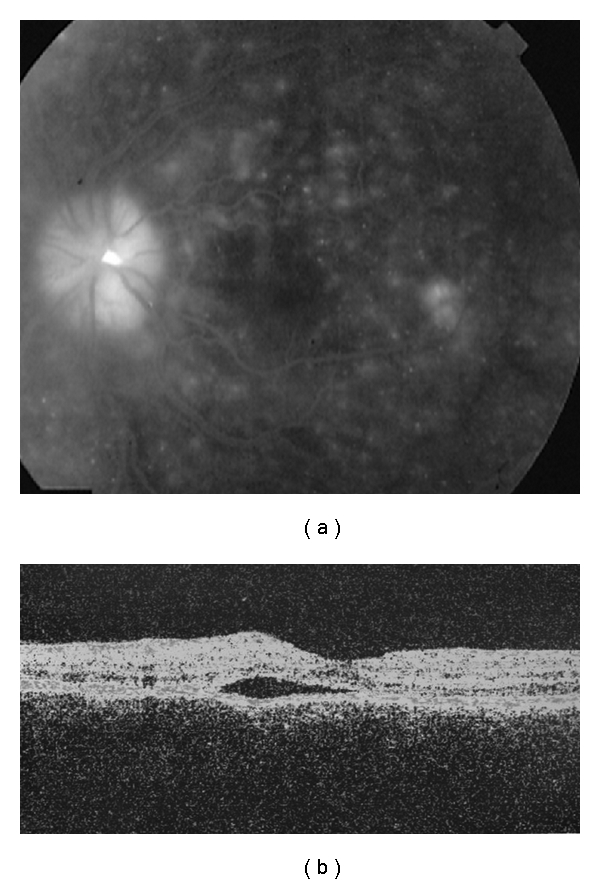
Images of the left eye at presentation. (a) Fluorescein angiography shows multiple, punctate, hyperfluorescent RPE changes and optic nerve hyperfluorescence. (b) Optical coherence tomography shows subretinal fluid in the macula.

A 50-year-old Hispanic woman presented with headaches and conjunctival injection for 14 days, and blurry vision with bilateral eye pain for 6 days. Vision was 20/400 in the right eye and 20/80 in the left eye. Anterior segment showed bilateral iritis. Vitritis was present. B-scan revealed choroidal thickening. Fluorescein angiography (FA) and ocular coherence tomography (OCT) showed subretinal fluid consistent with exudative RD (Figure 1; left eye). Lumbar puncture was normal. 

**Figure 2:**
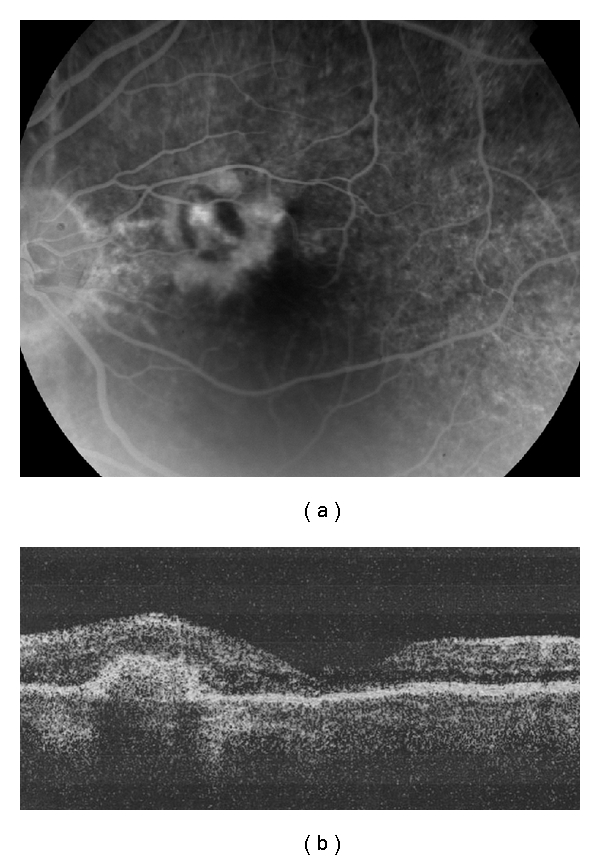
Images of the left eye after a 5-month loss to follow-up (the visual acuity was finger counting). (a) Fluorescein angiography shows an active CNVM. (b) Optical coherence tomography shows a parafoveal CNVM and mild intraretinal fluid.

**Figure 3:**
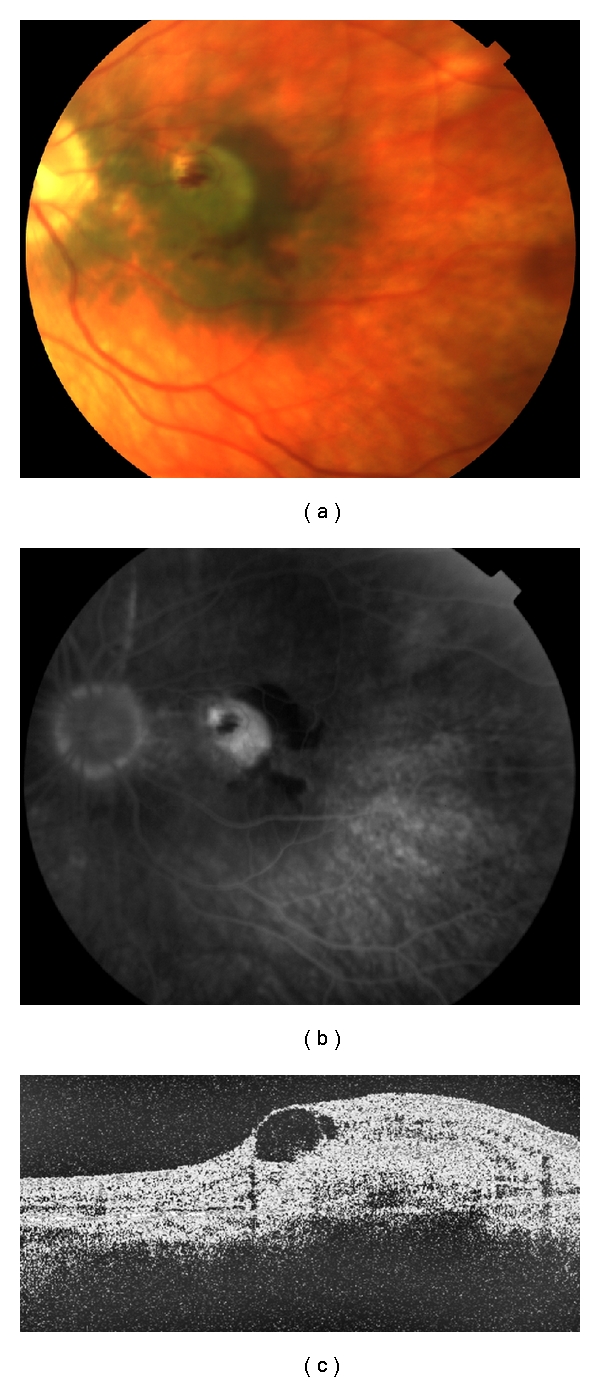
Images of the left eye 12 months after the third ranibizumab injection. (a) Color fundus photo shows retinal hemorrhage surrounding a CNVM, hyperpigmented macular RPE, and peripheral RPE hypopigmentation. (b) Fluorescein angiography shows a CNVM and hyperfluorescent, peripheral RPE changes. (c) Optical coherence tomography shows a parafoveal CNVM with a significantly increased amount of intraretinal fluid (compare with Figure 2(b)).

Diagnosis of VKH was established and ocular inflammation was initially treated with topical and systemic corticosteroids. After 14 days, inflammation subsided and vision improved to 20/40 in both eyes. Ten weeks later, because of morbidity associated with prednisone use and the chronicity of VKH, we initiated methotrexate (MTX) treatment. Visual acuity remained stable for 3 months. After a 5-month loss to follow-up (without immunosuppressive treatment for the last 2 months), the patient returned with recurred inflammation, 20/25 vision in the right eye, and finger counting (CF) vision in the left eye because of a parafoveal CNVM with mild intraretinal fluid (Figure 2). MTX treatment was reestablished. Following 3 intravitreal ranibizumab injections 6 weeks apart, vision improved to 20/40 in the left eye. Twelve months after the third injection, although the inflammation was controlled with MTX, vision in the left eye had again decreased to CF due to subretinal hemorrhage, increased amount of intraretinal fluid, and a recurrent CNVM (Figure 3). This was treated with a fourth intravitreal injection of ranibizumab. However, neither vision nor the CNVM showed improvement. The patient was ambivalent about receiving subsequent injections, and with a centrally located macular scar, the expected visual outcome was extremely poor. At last followup, 20 months after the fourth injection, vision in the left eye was 20/400 with pinhole and an inactive CNVM with RPE hypertrophy was noted. Visual acuity in the fellow eye was 20/30. 

## 3. Discussion

VKH may be complicated by the development of treatment-refractive CNVMs, which prognosticate a poor visual outcome [4]. Vascular endothelial growth factor (VEGF) is a key inducer of neovascularization [5]. Thus, ranibizumab, an anti-VEGF antibody, is a rational modality for managing CNVM formation. Anti-VEGF compounds have shown to be effective against idiopathic choroidal neovascularization (CNV) and CNV secondary to myopia, uveitis, angioid streaks, central serous chorioretinopathy, and punctate inner choroidopathy [6–9]. More specifically, Wu and colleagues [10] successfully treated 2 cases of CNV secondary to VKH with intravitreal injections of bevacizumab. Some additional treatment options that warrant investigation in the management of VKH-associated CNVMs include photodynamic therapy, laser photocoagulation, and combinational pharmacotherapy of anti-VEGF compounds with immunosuppressants. For example, in a study of subfoveal CNV in 6 VKH patients, Nowilaty et al. [11] showed that treatment with PDT with verteporfin was safe and effective. While Soheilian and coworkers [12] demonstrated that addition of MTX to bevacizumab in the treatment of CNV secondary to age-related macular degeneration may enhance CNV regression and decrease scar tissue formation by an unknown mechanism. 

In our patient, intravitreal ranibizumab initially led to CNVM regression as well as improvement and stabilization of vision. However, even in the absence of apparent inflammation, the CNVM recurred and became refractory to ranibizumab. Despite the fact that inflammation and neovascularization can be managed with immunomodulators, such as corticosteroids and MTX, and anti-VEGF compounds, the chronicity of this disease may lead to permanent loss of vision. In patients with VKH complicated by CNVM, prospective studies should be carried out to determine whether prolonged anti-VEGF therapy, alone or in combination with other treatment modalities, should be considered to reduce recurrent membrane formation, even in the absence of ocular inflammation.
